# The impact of electronic product use on children with tic disorders and ADHD, and management strategies: a review

**DOI:** 10.3389/fpsyt.2025.1665047

**Published:** 2025-10-21

**Authors:** Yi Zhang, Jiaqi Zhang, Xiaolu Yu, Jie Zhang, Yan Xu, Hongtao Cui

**Affiliations:** ^1^ Department of Pediatrics, First Affiliated Hospital, Henan University of Chinese Medicine, Zhengzhou, Henan, China; ^2^ School of Pediatrics, Henan University of Chinese Medicine, Zhengzhou, Henan, China; ^3^ Chongqing Hospital of Traditional Chinese Medicine, Chongqing, China

**Keywords:** attention deficit hyperactivity disorder (ADHD), tic disorders (TDs), electronic product use, management strategies, children, review

## Abstract

The pervasive use of electronic products raises significant neurodevelopmental concerns for children with Attention Deficit Hyperactivity Disorder (ADHD) and Tic Disorders (TDs), a vulnerable population particularly susceptible to the negative impacts of electronic products and at higher risk for problematic usage patterns. The clinical management of this issue is challenged by an incomplete understanding of the impact mechanisms. A review of the literature reveals these effects are complex and primarily functional, affecting systems like dopamine and executive functions, rather than causing widespread organic brain damage. Since the severity of the impact varies and absolute prohibition is often not the best approach, scientific management that focuses on content, duration, and usage patterns is essential. Specific, well-managed digital content may even have neutral or beneficial effects. Therefore, the paradigm for managing electronic product use must shift from simplistic restriction to scientific guidance and individualized strategies. This review offers an evidence-based framework to help clinicians tailor advice for each child’s developmental profile, moving beyond generic restrictions to foster healthy development in the digital age.

## Highlights

- Existing knowledge indicates widespread electronic product use among children with ADHD/TDs, often linked to negative impacts, with current guidance lacking nuance.
- This review reveals the impact is complex, primarily functional, and conditional, necessitating a shift from prohibition to scientific, individualized management focused on content, duration, and usage patterns.
- A key addition is the identification of potential benefits from specific, well-managed digital tools, challenging the “all-negative” view.
- For clinical practice and families, this study provides evidence-based principles for tailored screen management plans, moving beyond generic restrictions.
- For policy and future science, it underscores the need for clear guidelines, collaborative support systems, and further research into effective strategies and safer digital interventions.

## 1 Introduction

Electronic product use, a broad term encompassing interactions with devices such as smartphones, tablets, computers, various video games, and social media, has become deeply integrated into the daily lives of contemporary children and adolescents, serving as crucial tools for information acquisition, learning, leisure, and social interaction. In this review, the term “electronic products” specifically refers to screen-based digital media, a definition aligned with guidance from the World Health Organization (WHO) and the American Academy of Pediatrics (AAP) (1). Statistics indicate a continuous global increase in average daily screen exposure time among children and adolescents, a phenomenon particularly pronounced during the COVID-19 pandemic (2). While the proliferation of electronic products undoubtedly offers numerous conveniences and opportunities, their potential health risks, especially concerning the neurodevelopment of children and adolescents, are garnering increasing attention from parents, educators, and clinicians. For special pediatric populations with neurodevelopmental disorders such as Attention Deficit Hyperactivity Disorder (ADHD) and Tic Disorders (TDs), the impact of electronic product use and its management strategies are even more complex. This is particularly true as evidence links prolonged screen time to increased symptom severity in TDs (3), and identifies sedentary behavior—a major component of screen use—as a risk factor for ADHD (4).

ADHD is a common neurodevelopmental disorder in childhood, characterized by age-inappropriate inattention, hyperactivity, and impulsivity. TDs, including the more widely known Tourette Syndrome (TS), are primarily characterized by involuntary, rapid, repetitive motor and/or vocal tics. These two conditions not only severely affect a child’s academic achievement, social functioning, and emotional health but are also often accompanied by comorbid issues like anxiety, depression, and sleep disorders. In clinical practice, a general recommendation of “strict limitation” on electronic product use is often given for these children, primarily due to concerns that excessive screen exposure might exacerbate core symptoms, interfere with sleep, or induce other behavioral problems. However, such advice often lacks fine-grained, evidence-based guidance, leaving parents perplexed in specific management situations and potentially unnecessarily depriving children of opportunities to benefit from certain useful digital resources.

In recent years, an increasing number of studies have begun to focus on the relationship between electronic product use and the symptoms, cognitive functions, and overall well-being of children with ADHD/TDs. Preliminary evidence suggests that excessive electronic product use may be associated with increased severity or risk of relapse of TDs symptoms (3, 5). For children with ADHD, early screen exposure (e.g., at 1–3 years old), especially to educational and cartoon videos, has been found to be associated with an increased risk of preschool ADHD (6). Moreover, “problematic use” of digital media, rather than mere total electronic product use, has been shown to be a stronger predictor of the persistence or exacerbation of ADHD symptoms (7). Furthermore, the negative impact of electronic product use on sleep is well-documented (8–11), and sleep disturbance itself is a common comorbidity and symptom-worsening factor in both ADHD and TDs. Regarding cognitive function, some studies have indicated that electronic product use might be a negative predictor of cognitive performance, including executive functions (EFs), in children with ADHD (12).

Despite these findings, existing research still presents knowledge gaps or controversies in several key areas. (1) Unclear impact mechanisms: The specific neurobiological (e.g., how the dopamine system is affected (13) and psycho-behavioral mechanisms (e.g., specific pathways of sensory overload or executive function impairment) through which electronic products affect ADHD/TDs symptoms require more in-depth elucidation. Regarding whether they cause “organic brain damage,” direct evidence is currently lacking, with findings pointing more towards functional changes. (2) Vague boundaries for “moderate” use: There are currently no widely accepted guideline parameters (duration, frequency, content) for electronic product use specifically tailored for children with ADHD/TDs, and even general pediatric definitions, such as ≤1–2 hours of daily high-quality content, are difficult to implement (14). (3) Insufficient research on differential impacts of content and interaction modes: Different types of electronic screen content (e.g., educational software, video games, social media) and different usage modes (active participation *vs*. passive reception) may have significantly different effects on these children, and few studies examine the differential effects of content type versus total duration; for example, Wu found that interactive video content did not carry the same preschool ADHD risk as passive viewing in toddlers (6). (4) Inadequate exploration of potential benefits and positive guidance strategies: Research on whether specific types of electronic products (e.g., specially designed therapeutic games, assistive learning tools) might, under specific management and guidance, have neutral or even beneficial effects on children with ADHD/TDs, including improving specific cognitive functions or emotion regulation abilities (15–18) has begun but is far from adequate.

The rapid development of the digital information age has made electronic screens an indispensable part of learning, entertainment, and socialization for children and adolescents. However, this popularization is accompanied by growing concerns about potential health impacts, particularly for vulnerable groups during neurodevelopment, such as children with ADHD and TDs. ADHD, characterized by persistent inattention and/or hyperactivity-impulsivity, affects approximately 5-7% of school-aged children globally. TDs manifest as recurrent, involuntary motor or vocal tics, with TS being the most complex form, affecting about 0.3-1% of the population (19). These disorders not only cause learning difficulties, social impairments, and emotional distress for the affected children but also place a heavy burden on families and society. Against this backdrop, exploring the impact of electronic product use on children with ADHD and TDs holds significant practical importance. Current clinical practice often leans towards strict limitation or even prohibition of electronic product use for these children, based mainly on general concerns and scattered observations, lacking systematic evidence and detailed guidelines. Such a “one-size-fits-all” approach may not only be difficult to enforce but also overlook the potential positive roles of electronic products in education, cognitive training (e.g., serious games designed for ADHD (15, 20), and social connection. Therefore, a scientific and comprehensive assessment of the risks and potential benefits of electronic product use, along with clarification of its impact mechanisms, extent, and reasonable management strategies, is of crucial theoretical value and clinical guidance for optimizing comprehensive intervention plans, improving quality of life, and promoting healthy development in children with ADHD and TDs.

In recent years, international research on the relationship between electronic product use in children and adolescents and their neurodevelopment and mental health has proliferated. This growing attention is also reflected in multiple systematic and scoping reviews targeting this field. For instance, Peñuelas-Calvo systematically reviewed the use of video games in the assessment and treatment of ADHD (21), while Păsărelu revealed the significant gap between commercially available ADHD mobile apps and the evidence in scientific literature (22). Other reviews have focused on more specific technologies, such as mobile games (23), or have encompassed a broader range of emerging technologies including wearable devices and robots (24). Despite this, existing research remains deficient in the depth of mechanistic elucidation, the refinement of intervention strategies, and the tracking of long-term impacts. In particular, there is currently a lack of a single review that simultaneously integrates the evidence from both the ADHD and TD fields and distills a clear, actionable clinical management framework from the complex body of evidence.

For children with ADHD and TDs, studies have primarily focused on: (1) Association between electronic product use and symptom severity: Numerous cross-sectional and some longitudinal studies have explored the relationship between total screen exposure time, specific types of screen activities (e.g., video gaming, social media use), and core ADHD symptoms (inattention, hyperactivity/impulsivity) or tic frequency/intensity. Results often suggest an association between excessive use and symptom worsening (3, 6). However, the directionality of causality, specific impact thresholds, and differential effects of various screen content require further high-quality research. (2) Impact on comorbid problems: Research has also examined electronic product use in relation to common comorbidities in children with ADHD/TDs, such as sleep disorders, anxiety/depressive mood, and the risk of problematic internet use/gaming disorder. Substantial evidence indicates that electronic product use, especially at night, significantly interferes with sleep (10, 11), and sleep problems, in turn, can exacerbate ADHD/tic symptoms. Some studies also suggest that excessive screen exposure may increase the risk of internalizing emotional problems and internet addiction (8, 25). (3) Exploration of digital interventions: Distinct from mere restriction, some studies are exploring the use of specially designed applications, video games (serious games, exergames), or virtual reality technology as adjunctive therapeutic or training tools for ADHD or TDs, showing preliminary potential in improving specific symptoms or cognitive functions (15, 17, 20).

Beyond clinical observations, other research has explored underlying mechanisms: (1) Impact on cognitive functions: Particularly for children with ADHD, the effect of electronic product use on EFs (e.g., working memory, inhibitory control, cognitive flexibility) is a research hotspot. Some studies have observed negative associations (12), while others have explored the potential of using specific cognitive training games to improve EFs (16, 18). (2) Exploration of potential impact mechanisms: Neuroimaging and neurobiochemical studies are beginning to investigate the mechanisms by which screen activities affect brain function and behavior, such as involvement of the dopamine reward pathway (13), brain connectivity patterns, and effects on sensory processing; however, research in this area is still in its nascent stages. Overall, current research recognizes the complex impact of electronic product use on children with ADHD/TDs and has explored it from various perspectives. Nevertheless, existing studies are still deficient in the depth of mechanistic elucidation, refinement of intervention strategies, and long-term impact tracking. There is a particular lack of in-depth comparative analysis concerning different types of electronic content, usage patterns, and individual differences.

Given this context, this review aims to conduct an in-depth exploration of the following four core questions by systematically reviewing and analyzing relevant scientific literature from the PubMed database: First, is the current recommendation for children with tic/hyperactivity disorders to “not watch” electronic products absolute? Should restrictions apply to all electronic products, or to specific content or durations? Second, what are the specific mechanisms by which electronic products affect these children? Do they cause organic brain damage? Which brain functions or regions are primarily affected? Third, what is the extent of this impact? Is it merely a slight exacerbation of symptoms, or can it lead to more profound negative effects? Fourth, if not entirely prohibited, how should the boundary for “moderate” use be scientifically defined and managed? Is it possible that, under certain conditions, some electronic products could be beneficial or have a neutral impact? By addressing these questions, this review aims to provide clinicians, educators, and parents of affected children with a more comprehensive, nuanced, and evidence-based understanding, thereby promoting scientific, individualized management of electronic product use in children with Attention Deficit Hyperactivity Disorder (ADHD) and Tic Disorders (TDs), and ultimately improving their health outcomes and quality of life.

To achieve these aims, this review draws upon an extensive synthesis of literature primarily sourced from the PubMed database, supplemented by key theoretical papers and influential reviews in the field. This review will also specifically note how the included studies address comorbidity, indicating whether children with co-occurring ADHD and TDs are analyzed as a distinct subgroup. The literature selection prioritized high-quality research and significant contributions relevant to understanding the complex interplay between electronic product use and neurodevelopmental outcomes in children with ADHD and TDs, as well as those exploring effective impact mechanisms and management strategies. This diverse body of knowledge is synthesized herein to offer a comprehensive overview, identify critical gaps, and propose a novel framework for guidance.

## 2 Methodology

### 2.1 Search strategy

This narrative review is primarily based on a systematic search of the PubMed database. PubMed was selected as the core information source for several key reasons: (1) The central questions of this review are distinctly biomedical in nature, covering neurobiological mechanisms, clinical symptomatology, and intervention strategies, for which PubMed offers the most comprehensive and timely coverage; (2) PubMed’s unique MeSH (Medical Subject Headings) system and its clinical queries filters facilitate the construction of a structured, transparent, and highly reproducible search strategy; and (3) While other databases such as PsycINFO and Embase contain relevant literature, a strategy of “core PubMed search plus citation tracking” was deemed to achieve an optimal balance between ensuring coverage of key literature and resource utility, given the clinical focus of this review and the significant overlap among these databases.

The search was limited to articles published from January 2010 to May 2025 to ensure the inclusion of research from the past 15 years, a period of rapid digital media evolution. The search was restricted to the English language. The search strategy combined MeSH terms and free-text keywords, with the main search term clusters as follows:

Disease-related terms: (“Attention Deficit Disorder with Hyperactivity”[Mesh] OR “Tic Disorders”[Mesh] OR “Tourette Syndrome”[Mesh]) AND (“ADHD” OR “tic” OR “Tourette”).
Exposure-related terms: (“Video Games”[Mesh] OR “Internet”[Mesh] OR “Social Media”[Mesh] OR “Cell Phone”[Mesh]) AND (“electronic product use” OR “digital media” OR “electronic product” OR “video game” OR “internet use” OR “smartphone”).
Boolean Logic: The two sets of terms were combined using the ‘AND’ Boolean operator.

### 2.2 Study selection and data extraction

**Figure 1:**
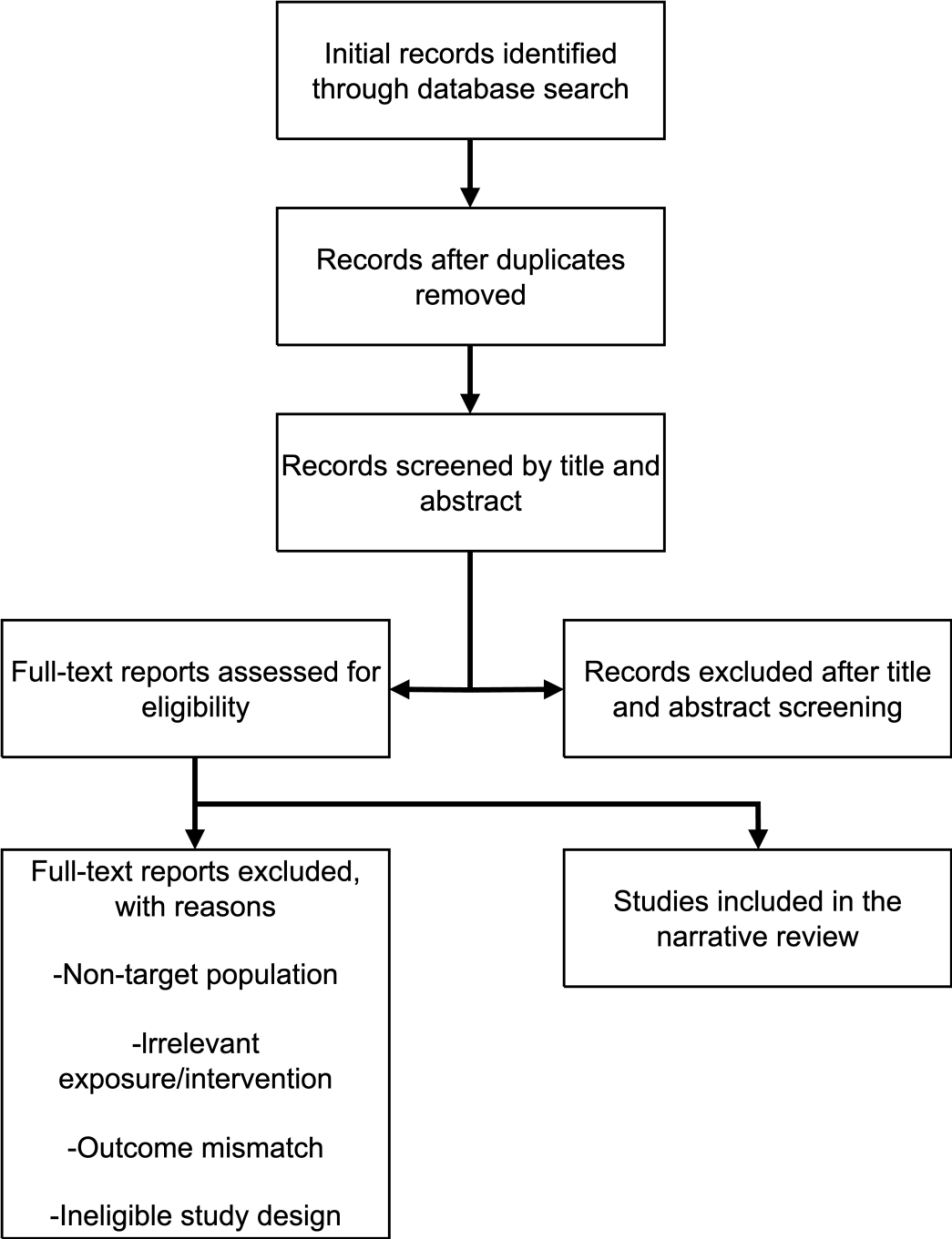
Flow diagram for study selection.

The study selection process as detailed in the flowchart (**Figure 1**). Inclusion criteria were: (1) studies involving children and adolescents (aged <18 years) with a confirmed diagnosis of ADHD and/or TDs; (2) studies investigating any form of electronic product use as an exposure or intervention factor; (3) studies reporting outcome measures related to symptoms, cognition, sleep, behavior, neurobiological mechanisms, or quality of life; and (4) study types including systematic reviews, meta-analyses, randomized controlled trials (RCTs), cohort studies, case-control studies, and cross-sectional studies. Exclusion criteria were: case reports, conference abstracts, and studies not directly related to the core topic.

Two researchers independently screened the literature and extracted data, with results cross-checked. Any discrepancies were resolved through discussion. Key information extracted included: author/year, study design, sample characteristics (size, age, diagnosis, medication status), methods for assessing electronic product use, key findings, and study limitations.

### 2.3 Quality assessment

This review acknowledges the importance of assessing the quality of included studies. However, as a narrative review aimed at integrating and providing a comprehensive overview of the existing evidence landscape, rather than conducting a meta-analysis, we did not perform a systematic, quantitative rating for each original study using tools such as the Newcastle-Ottawa Scale (for observational studies) or AMSTAR-2 (for systematic reviews) to be presented in a table. Nevertheless, in the synthesis and discussion of the evidence, we prioritized findings from studies with more rigorous designs (e.g., longitudinal studies, RCTs), larger sample sizes, and those published in high-quality journals. The design types of key studies have also been noted within the text.

## 3 Results

This section synthesizes the evidence regarding the impact of electronic product use on children with ADHD, TDs, and comorbid conditions. The findings are organized by diagnostic group to prevent conflation, and within each, are subdivided into negative effects, potential benefits, and proposed mechanisms. Key characteristics of the primary studies informing this synthesis are summarized in **Table 1**, while **Table 2** provides a comparative overview of the risks and benefits associated with different types of screen content.

**Table 1:** Summary of key studies on the impact of electronic product use in children with ADHD/TDs.

Author/year (PMID)	Study design	Sample information	Key findings	Main limitations
Ahmed et al. (2025) (3)	Retrospective case-control study	342 children with TDs, 270 healthy controls	Children with TDs had longer durations of electronic product use, which was significantly and positively correlated with tic severity (YGTSS).	Retrospective design; unable to determine causality.
Wu et al. (2025) (6)	Large-scale cross-sectional study	41,494 toddlers aged 1-3	Passive screen viewing in early childhood was associated with preschool ADHD risk in a dose-dependent manner; interactive videos showed no such association.	Relies on parent self-report; cross-sectional association.
Swisher et al. (2024) (11)	Cross-sectional study	192 children with TS, 192 healthy controls	Daily electronic product use ≥2 hours was significantly associated with poorer bedtime routines in children with TS.	Relies on parent self-report; cross-sectional design.
Dong et al. (2025) (12)	Cross-sectional study	184 children with ADHD	electronic product use was a negative predictor of cognitive function (based on PASS theory) in children with ADHD.	Cross-sectional design; did not differentiate content types; no control group.
Thorell et al. (2024) (7)	Systematic review	28 longitudinal studies	“Problematic use,” rather than total duration, has a stronger reciprocal and cyclical risk relationship with ADHD symptoms.	High heterogeneity among included studies.
Raz et al. (2024) (13)	Experimental study (with replication)	20 (Exp 1) & 36 (Rep) children with TDs	Viewing suspenseful movies and playing reward-based video games immediately increased tic frequency, possibly related to dopamine release.	Small sample size; short-term effects in a lab setting.
Rotstein et al. (2024) (17)	Randomized crossover trial	35 children with TDs	A gamified behavioral intervention (XTics) effectively prolonged inter-tic intervals and reduced tic severity.	Small sample size; relied on objective metrics more than clinical scales.
Benzing & Schmidt (2019) (15)	RCT	51 children with ADHD	An 8-week exergaming intervention improved inhibitory control, task-switching, and motor skills in children with ADHD.	Relatively small sample size; wait-list control group.
Gabarron et al. (2025) (18)	Systematic review of systematic reviews	26 systematic reviews (34,442 participants)	Digital interventions (games, VR, etc.) showed positive effects for ADHD, but evidence quality was generally low and side effects were underreported.	Varied quality of included reviews; risk of publication bias.

**Table 2:** Risks and potential benefits of different types of electronic content for children with ADHD/TDs.

Content type	Likely mechanisms	Primary impact direction	Strength of evidence
Passive Viewing (e.g., low-quality videos, cartoons)	Displaces developmental activities (sleep, exercise); low cognitive load; may entrench passive learning patterns.	Negative	Moderate to High
Fast-Paced/High-Stimulation Games (e.g., action, competitive games)	Over-activation of dopamine reward pathways; sensory overload; reinforces immediate gratification.	Negative (symptom exacerbation, addiction risk)	Moderate
Educational/Cognitive Training Apps (non-prescription)	Targeted cognitive skill practice (e.g., EFs); provides structured learning.	Neutral to Positive (highly dependent on quality and usage)	Low to Moderate
Exergaming (e.g., motion-sensing games)	Combines physical activity with cognitive engagement; improves motor coordination and EFs.	Positive	Moderate
Digital Therapeutics (e.g., EndeavorRx, XTics)	Gamified, clinically-validated cognitive or behavioral interventions; modulates specific neural functions.	Positive (as adjunctive therapy)	Moderate to High
Social Media (e.g., short-form videos, social platforms)	Social comparison; risk of cyberbullying; sleep deprivation; provides peer support (if guided).	Negative (unsupervised)/Neutral (supervised)	Moderate
Creative Applications (e.g., coding, drawing, music)	Fosters active thinking and creativity; develops logical thinking and artistic expression.	Positive	Low (theoretical and anecdotal)

Strength of evidence is estimated based on the type and volume of studies included in this review (High: based on systematic reviews/meta-analyses or multiple RCTs; Moderate: based on RCTs or high-quality longitudinal/cross-sectional studies; Low: based on preliminary studies, small-sample trials, or theoretical inference).

### 3.1 The impact of electronic product use on children with ADHD

#### 3.1.1 Negative effects

A substantial body of evidence, largely from observational studies, links excessive or problematic electronic product use to a range of negative outcomes in children with ADHD, including symptom exacerbation, cognitive impairment, sleep disruption, and a heightened risk for behavioral addictions.

The link between electronic product use and ADHD symptoms is well-documented. For instance, a large-scale cross-sectional study on toddlers found that increased daily screen time, particularly watching educational and cartoon videos, was associated with an elevated risk of preschool ADHD in a dose-dependent manner (6). Critically, a systematic review which included 28 longitudinal studies, concluded that it is “problematic use”—characterized by loss of control—rather than total time of electronic product use alone, that demonstrates a stronger reciprocal and potentially vicious cycle with ADHD symptoms (7). This suggests a complex interplay where symptoms may drive problematic use, which in turn exacerbates symptoms. Furthermore, children with ADHD are particularly vulnerable to developing behavioral addictions. Multiple reviews and case-control studies provide strong correlational evidence that ADHD is a significant risk factor for developing Problematic Internet Use (PIU) and Internet Gaming Disorder (IGD). Impulsivity and emotional dysregulation, core features of ADHD, play important roles in this vulnerability (25). Further research has specified that this risk is especially high for the combined and predominantly hyperactive/impulsive subtypes of ADHD (26).

Cognitive function, particularly Executive Functions (EFs), may also be adversely affected. A cross-sectional study identified electronic product use as a negative predictor of cognitive function (including core EF components like planning and attention) in children with ADHD (12). However, it is crucial to interpret this correlational finding with caution, as it cannot establish causality. It remains unclear whether excessive electronic product use impairs EFs, or if children with pre-existing EF deficits are more drawn to screen-based activities.

Finally, the negative impact on sleep is robustly documented. A path analysis model has proposed a causal pathway, suggesting that screen exposure indirectly exacerbates ADHD symptoms by increasing sleep problems (10). This is supported by direct associational evidence which confirmed that nighttime electronic product use in adolescents with ADHD was significantly linked to sleep problems, daytime sleepiness, and internalizing symptoms (8, 9).

#### 3.1.2 Potential benefits

A comprehensive review, for instance, concluded that various emerging technologies—including mobile health, wearables, and serious games—show promising implications for supporting self-regulation in children with ADHD (24). Focusing specifically on video games, a systematic review found that game-based therapeutic interventions were generally effective in improving cognitive areas and decreasing ADHD symptoms (21). This distinction is evident even in early childhood. The same study that found a negative link with passive viewing also reported that electronic product use involving interactive videos showed no such significant association with ADHD risk (6). This finding provides a crucial insight: active, interactive screen activities may have different neurocognitive effects compared to passive consumption.

Building on this, a growing body of research explores digital tools as adjunctive therapies. A systematic review of reviews evaluated various digital interventions (including video games and VR) and noted their positive effects in improving attention and EFs, though it also cautioned about low evidence quality and under-reported side effects (18). More concrete causal evidence comes from specific Randomized Controlled Trials (RCTs). For example, an 8-week exergaming intervention was shown to improve specific EFs (e.g., inhibition and switching) and motor skills (15). Similarly, AI-driven digital cognitive stimulation programs like KAD_SCL_01 have demonstrated efficacy in improving inhibitory control (16), and digital therapeutics such as AKL-T01 have been shown to improve clinical outcomes and reflect real-time cognitive changes (27). These studies collectively indicate that carefully designed applications, under professional guidance, can become beneficial components of a comprehensive intervention plan.

#### 3.1.3 Proposed mechanisms

The mechanisms underlying these effects are thought to be primarily functional, affecting neurotransmitter systems and cognitive networks. The evidence for these mechanisms is largely correlational and theoretical, often inferred from studies on related conditions like IGD.

A leading hypothesis involves the dysregulation of the brain’s dopamine reward system. Electronic products, especially highly stimulating video games, can strongly activate dopaminergic pathways through instant feedback and unpredictable rewards. One review mentioned that this activation may affect ADHD-related behaviors (28). While direct causal evidence is limited, the strong, consistent association between ADHD and IGD, a condition linked to dopamine system dysfunction, provides compelling indirect support for this mechanism (25, 29).

Another key mechanism is the interference with the development and functioning of EFs and their underlying neural basis in the prefrontal cortex. The prolonged passive information reception, frequent task-switching, and instant gratification common in general electronic product use can reduce opportunities for deep thinking and delayed gratification, thereby hindering EF development. This presents another contradiction that requires careful consideration: while uncontrolled screen exposure appears to tax or impair EFs (12), some targeted, screen-based cognitive training software is specifically designed with the goal of improving these same functions (30). This reinforces the conclusion that the nature of the digital interaction, rather than the screen itself, is the critical determinant of the outcome.

### 3.2 The impact of electronic product use on children with tic disorders

#### 3.2.1 Negative effects

For children with Tic Disorders (TDs), a significant body of research links electronic product use to the direct exacerbation of tic symptoms and associated impairments such as sleep disruption. Strong correlational evidence from a retrospective case-control study clearly indicated that children with TDs had significantly longer durations of electronic product use than healthy controls, and this duration was positively correlated with tic severity as measured by the Yale Global Tic Severity Scale (YGTSS), with daily electronic product use acting as an independent predictor of the total score (3). Further supporting this, another study identified average daily electronic product use as a poor prognostic factor for symptom relapse after medication withdrawal (5), and early observations also noted that activities like playing video games and watching television could exacerbate tic symptoms (31). Moving beyond association, experimental research provided direct, short-term causal evidence, demonstrating that watching suspenseful movies or playing reward-based video games immediately and significantly increased tic frequency in a laboratory setting (13). In addition to the direct impact on tics, sleep is also a key area of concern. A cross-sectional study found that daily electronic product use of two hours or more in children with Tourette Syndrome was significantly associated with poorer bedtime routines and shorter total sleep duration, which in turn can negatively impact tic control (11).

#### 3.2.2 Potential benefits

In direct contradiction to the negative effects of general electronic product use, specifically designed and targeted digital interventions are emerging as a promising therapeutic avenue for TDs, underscoring the critical distinction between unregulated screen consumption and prescribed digital tools. The most compelling causal evidence comes from a randomized crossover trial on XTics, a gamified behavioral intervention, which found that this screen-based tool effectively prolonged inter-tic intervals and reduced tic severity by reinforcing tic suppression behavior (17). Furthermore, digital technology has proven effective in increasing access to established therapies. Studies have shown that internet-delivered Cognitive Behavioral Therapy (iCBT), such as Exposure and Response Prevention (ERP), can be successfully applied to children with Tourette Syndrome and chronic TDs, significantly improving treatment accessibility for these evidence-based interventions (32, 33).

#### 3.2.3 Proposed mechanisms

The mechanisms underlying the impact of electronic products on TDs are thought to relate primarily to physiological arousal, sensory processing, and the modulation of motor control circuits, with evidence for these pathways being largely inferred, experimental, or theoretical. The immediate increase in tics observed in an experimental study was hypothesized by its authors to be related to the phasic release of striatal dopamine, which is linked to the emotional excitement and reward anticipation inherent in engaging content; this heightened arousal may lower the threshold for tic expression (13). Another theoretically proposed mechanism, for which direct research is still needed, is sensory overload. Given that children with TDs often exhibit sensory processing sensitivity, it is plausible that the high-intensity, rapidly changing visual and auditory stimuli from fast-paced games and videos can overwhelm their nervous system’s processing capacity. This may lead to a dysregulation of sensorimotor gating and a subsequent increase in tic frequency. Finally, the disruption of the sleep-wake cycle, as suggested by correlational data, represents a more indirect mechanism, whereby poor sleep quality resulting from electronic product use directly exacerbates core symptoms, including tic frequency and intensity (11, 34).

### 3.3 Impact on children with comorbid ADHD and tic disorders

For children diagnosed with both ADHD and TDs, the clinical presentation, functional impairment, and response to environmental factors are often more complex. While research explicitly isolating this comorbid group is scarce, a synthesis of evidence reveals a landscape of synergistic risks and unique management challenges.

#### 3.3.1 Negative effects

For children with ADHD+TDs, inherent neurobiological vulnerabilities may be significantly amplified by electronic products, creating a synergistic negative effect where the combined risk exceeds the sum of its parts. This is most evident in the markedly increased risk of developing Problematic Internet Use (PIU) and Internet Gaming Disorder (IGD). Given that ADHD is already a primary risk factor for IGD (35) (36), the co-occurrence of TDs—with its associated sensory sensitivities and stress-coping mechanisms—appears to constitute a powerful diathesis for addictive online behaviors, as suggested by a study where tic disorders were a common comorbidity in internet-addicted adolescents (37). This heightened vulnerability is reflected in a poorer clinical prognosis; a three-year longitudinal study highlighted that individuals with IGD and comorbid ADHD had significantly lower recovery rates, suggesting that unresolved neurodevelopmental symptoms act as strong maintaining factors against recovery (38). Furthermore, these children face a compounded burden on sleep, where the risks independently associated with both disorders are exacerbated by the direct negative impact of electronic product use, creating a vicious cycle of sleep deprivation that can worsen both ADHD and TD symptoms (39).

#### 3.3.2 Potential benefits

When considering digital interventions for the ADHD+TDs group, a more cautious approach is warranted, as a core clinical dilemma emerges from a potential trade-off between symptom domains. While, in theory, cognitive training games for ADHD or gamified behavioral interventions for TDs could be beneficial, their design elements may create conflicts. For example, a cognitive training game designed to improve executive functions in ADHD, with its high-stimulation elements such as rapid responses and time pressure, might inadvertently exacerbate tic symptoms by increasing cognitive load and physiological arousal, a phenomenon observed in clinical case studies (40). Therefore, selecting digital tools for this population requires careful individual assessment, prioritizing applications with controllable stimulation levels that can accommodate the characteristics of both conditions, alongside close monitoring during use.

#### 3.3.3 Proposed mechanisms

The shared neurobiological underpinnings of ADHD and TDs provide a theoretical framework for these synergistic effects, with two non-exclusive mechanisms being hypothesized. The first is the Cortico-Striatal-Thalamo-Cortical (CSTC) Circuitry Common Pathway Hypothesis, where exogenous hyper-stimulation from gaming is proposed to act as a “common pathway” disruptor for the circuitry implicated in both disorders, simultaneously exacerbating the impulse control failures of ADHD and the motor inhibition difficulties of TDs (41). The second is the Cognitive-Motor Resource Competition and “System Overload” Hypothesis, which posits that the dual demands of complex games deplete the finite cognitive resources needed for top-down control of both tics and impulses. This can lead to a “system overload” of the cognitive control system, resulting in a disinhibition phenomenon that manifests as a worsening of both hyperactive/impulsive behaviors and tics (40).

### 3.4 Variability in impact severity and moderating factors

The severity of the impact of electronic products on children with tic/hyperactivity disorders is not uniform but exhibits significant individual differences and situational dependency. The scope of impact can range from minor daily symptom fluctuations to profound negative effects on cognition, emotion, sleep, social interaction, and even overall lifestyle. Understanding the variability in these impact severities and their underlying moderating factors is crucial for developing individualized management strategies.

At the symptom level, the degree of impact can be dynamic. Research by Ahmed (3) showed a quantitative association between electronic product use and tic severity, while Wu study (6) revealed a dose-dependent relationship between screen exposure in early childhood and the risk of Attention Deficit Hyperactivity Disorder (ADHD), both confirming that duration of use is an important factor in impact severity. Exposure to specific media content (including high-suspense movies or reward-based games (13) can instantaneously increase tic frequency. Long-term excessive use is associated with persistent symptom worsening or high relapse rates aftertreatment (2, 5).

Beyond core symptoms, inappropriate use of electronic products can lead to broader and more profound adverse effects. In the cognitive domain, persistent screen exposure may impair key cognitive abilities such as executive functions (EFs) in children with ADHD, thereby affecting their academic performance and long-term adaptability (12). In terms of sleep health, chronic sleep deprivation and circadian rhythm disturbances not only exacerbate core symptoms but may also trigger a series of secondary health problems, including mood disorders, metabolic abnormalities, and decreased immune function (11, 34). At the behavioral level, children with ADHD/tics, due to their neurobiological characteristics, are more prone to developing Problematic Internet Use (PIU) or Internet Gaming Disorder (IGD) after excessive use of electronic products; these are behavioral addictions with significant social functional impairment (25, 26). Emotionally and socially, problematic social media use may not only be associated with anxiety and depressive moods but also expose them to risks such as cyberbullying (8, 42). Finally, inappropriate electronic product use habits are often part of an overall unhealthy lifestyle, frequently accompanied by insufficient physical activity and irregular routines, posing challenges to children’s comprehensive development (43, 44).

However, the severity of the aforementioned impacts is complexly modulated by multiple factors:

1. Individual factors: The child’s age and developmental stage are primary considerations. The brains of young children are particularly sensitive to environmental input, and excessive screen exposure in early childhood (e.g., 1–3 years old) may have more lasting potential impacts on neurodevelopment (6, 45). Furthermore, the severity of the child’s own disorder, the presence of comorbidities (e.g., anxiety disorder, depression, learning disabilities, sensory processing disorders), and their individual physiological and psychological vulnerabilities (e.g., impulse control ability, emotion regulation capacity, tolerance to specific sensory stimuli) will significantly modulate their response patterns and the extent of impact from electronic product stimuli.
2. Electronic product-related factors: First is the difference in content type. Wu et al.’s research (6) suggests that highly interactive video content may have a different impact on ADHD risk in young children compared to passively watched educational/cartoon videos. Raz et al.’s study (13) also highlighted the immediate impact of specific content elements (e.g., suspense, instant rewards) on tic symptoms. Generally, content that is educational, creative, highly interactive, moderately paced, and free of violence or excessive stimulation may pose a relatively lower negative risk. Second is the duration and frequency of use; most studies support a “dose-effect” relationship, meaning the longer the exposure time and the higher the frequency, the greater the potential risk. Third is the usage pattern; a review by Thorell (7) emphasized that “problematic use” (characterized by loss of control, compulsivity, and functional impairment) is a better predictor of adverse outcomes than total electronic product use alone. Finally, the usage context, such as prolonged solitary use in the bedroom versus moderated interactive use with parental companionship, has vastly different impacts; pre-sleep use is particularly disruptive to sleep.
3. Environmental factors: The family environment and parenting styles play crucial roles. Parents’ electronic product use habits (role modeling), rules for managing children’s electronic product use (whether there are clear limits, whether they are enforced), the quality of parent-child communication (whether electronic product use plans are discussed and negotiated with the child), and the ability to provide a rich variety of alternative activities (e.g., outdoor sports, parent-child reading, artistic development) profoundly affect children’s electronic product use behavior and its consequences. Güzel research (46)pointed out the association between mothers’ negative media role modeling and neglect of their children’s media use, and the development of problematic use in those children. Additionally, the school environment, peer influence, and broader socio-cultural factors may also indirectly modulate the extent of electronic products’ impact on children.

**Figure 2:**
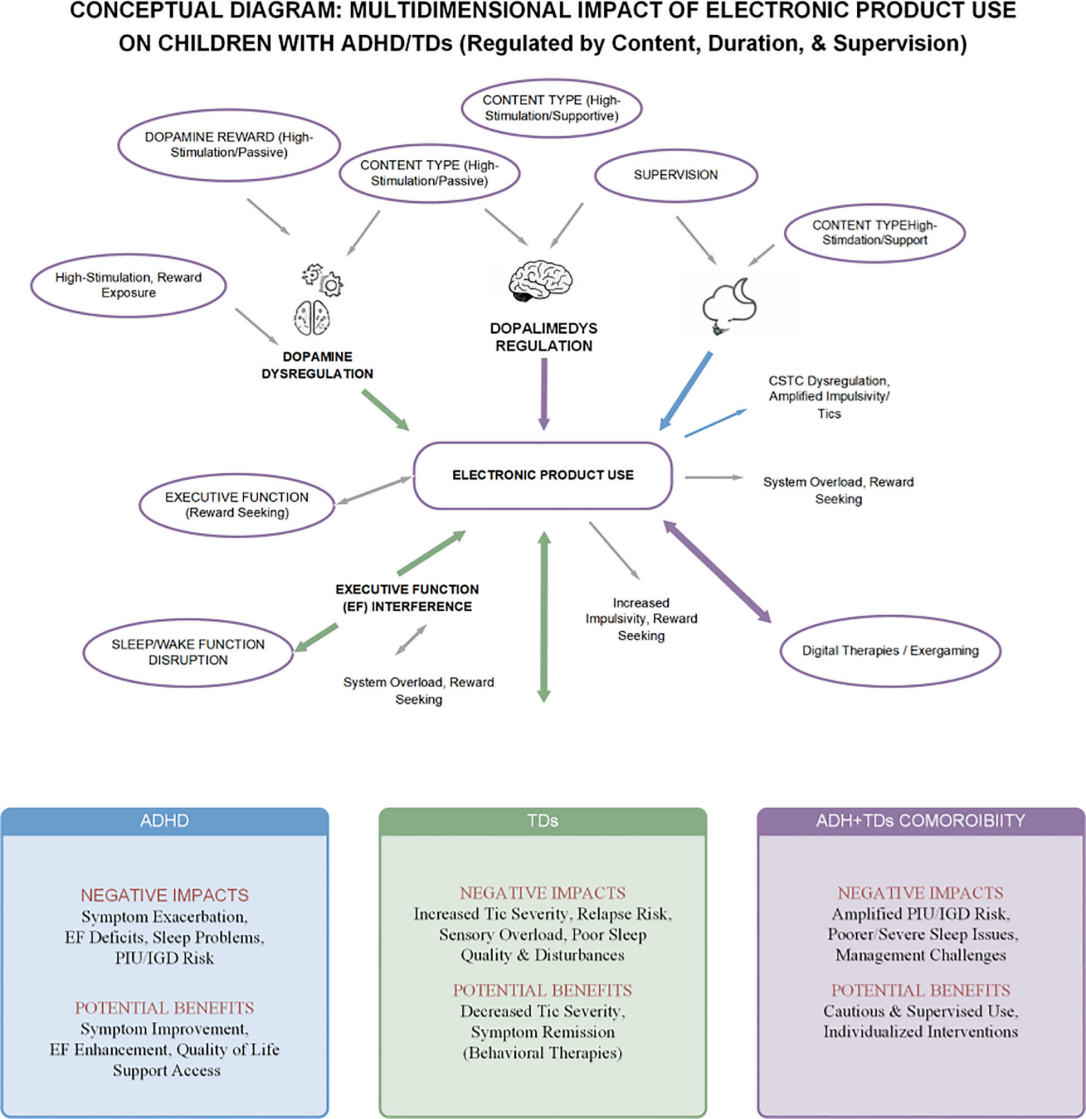
An ecological framework for electronic product use in neurodevelopmental disorders.

Therefore, when assessing the impact of electronic products on children with tic/hyperactivity disorders, an ecological, multifactorial perspective must be adopted. Fully considering the dynamic interactions among the individual, the product, and the environment is essential to more accurately determine the nature and severity of the impact and to formulate truly individualized and effective management and intervention strategies. **Figure 2** presents a conceptual model that visually synthesizes the complex relationships discussed in this chapter.

## 4 Scientific basis for defining “moderate” use and management strategies

After fully recognizing the complex potential impacts and the key moderating factors detailed in the previous chapter, the core issue naturally shifts from analysis to action: how to scientifically define and manage electronic product use to maximize potential benefits while mitigating known risks. A simplistic prohibition strategy is often impractical and may prevent children from accessing beneficial digital opportunities. Conversely, a laissez-faire approach heightens the risks of symptom exacerbation and behavioral addiction. Therefore, establishing a balanced, individualized framework for “moderate” use is crucial. This chapter synthesizes the evidence into actionable guiding principles for clinicians, parents, and educators.

### 4.1 Exploring the “degree”: evidence-based recommendations

Defining a “moderate” range is a dynamic and multidimensional issue. By integrating general health guidelines with the risk factors and content analyses discussed previously, we can outline key evidence-based recommendations.

First, general pediatric guidelines provide a valuable baseline. The 24-Hour Movement Behavior Guidelines, which recommend limiting recreational electronic product use to under 2 hours daily for school-aged children, serve as a sound starting point, as adherence is linked to better outcomes in children with ADHD (47, 48). However, this baseline must be adapted, as specific “warning lines” from research suggest stricter boundaries for certain populations, such as younger children or those with sleep disturbances (6, 11). Second, as the evidence synthesized in the previous chapter makes clear, focusing solely on “duration” is insufficient. The nature of the content (‘what to watch’) and the context of use (‘how to watch’) are equally, if not more, critical determinants of the outcome (6, 13). Therefore, management must prioritize content selection.

(1) Priority should be given to: Validated educational and cognitive training applications; Interactive and creative content that requires active engagement; Moderately paced, low-stimulation content; and Guided activities that promote positive social interaction.

(2) Strict limitation or avoidance should apply to: Content with excessive violence or addictive mechanics; low-quality passive entertainment; and unrestricted social media use that may induce anxiety or cyberbullying.

Furthermore, the context of use directly influences its “moderation.” Based on robust evidence, light-emitting screens should be avoided 1–2 hours before bedtime to protect sleep quality (8, 10). Best practices also include keeping devices in common family areas and encouraging parental co-engagement to transform use into an opportunity for interaction and guidance.

In conclusion, defining the “degree” of use is a dynamic balancing process, requiring flexible judgments based not only on duration but also on the quality of content, mode of interaction, and context of use, all tailored to each child’s specific situation.

### 4.2 Construction of individualized management strategies

Given the significant heterogeneity among children with Attention Deficit Hyperactivity Disorder (ADHD) and Tic Disorders (TDs) concerning symptom presentation, cognitive characteristics, comorbid conditions, and family environments, management strategies for electronic product use must emphasize individualization and dynamic adjustment. A universal “standard plan” is unlikely to adequately address the needs of all affected children; scientific management should be founded on a comprehensive assessment of individual characteristics and integrate multidimensional intervention elements.

Individualized assessment is a prerequisite for formulating management strategies and should encompass the following aspects:

1. Age and developmental stage: Children across different age groups vary in their comprehension of screen content, self-control capabilities, and the proportion of screen activities within their developmental requirements. For young children, management primarily focuses on strict limitations on total duration and passive viewing content. In contrast, for adolescents, the emphasis shifts more towards cultivating media literacy, identifying online risks, and balancing online and offline activities.
2. Core symptom characteristics and severity: A detailed evaluation of core ADHD symptoms (e.g., specific manifestations of inattention, degree and context of hyperactivity/impulsivity) and TD characteristics (e.g., tic type, frequency, intensity, and associated discomfort) is necessary. For children with severe visually-induced tics, more stringent consideration of technical parameters of screen content, such as frame rate and flicker, may be warranted.
3. Comorbid conditions: It is imperative to assess for the presence of comorbidities such as anxiety disorder, depression, learning disabilities, sleep disorders, and sensory processing abnormalities. These comorbidities can not only heighten the risk of inappropriate electronic product use (e.g., using games to escape real-life stress, prolonged nighttime electronic product use due to sleep problems) but may also amplify the negative impact of certain screen content (e.g., frightening information, social pressure).
4. Cognitive function level, particularly executive functions (EFs): Understanding a child’s strengths and weaknesses in areas like working memory, inhibitory control, planning and organization, and cognitive flexibility is instrumental in judging their adaptability to complex screen tasks and identifying potential cognitive training needs.
5. Interests, strengths, and needs: Management is not solely about restriction; children’s reasonable interests and developmental needs must also be taken into account. Consideration of children’s legitimate interests and developmental requirements is equally vital. If a child demonstrates a strong interest in programming or digital art, this can be incorporated as part of their electronic product use under guidance, thereby fostering the development of relevant skills.
6. Family environment and support system: This involves assessing parents’ media literacy, management capabilities, available time for companionship, consistency among family members regarding electronic product use rules, and the overall family atmosphere (e.g., quality of parent-child relationships, stress levels). Based on this individualized assessment, the construction of a multidimensional management plan should incorporate the following elements:
  1. Refined time management: This extends beyond merely setting upper limits for total daily/weekly electronic product use (e.g., referencing general guidelines and adjusting based on individual circumstances) to, more crucially, establishing “electronic product use scenario rules.” For instance, specific times such as learning periods, homework sessions, mealtimes, outdoor activity periods, and 1–2 hours before bedtime should be clearly designated as “screen-free” or “low-screen” periods. The use of timers, parental control software, and other tools to aid enforcement should be encouraged. The primary objective is to ensure that electronic product use does not unduly displace other activities essential for child development, such as adequate sleep, physical exercise, face-to-face social interaction, and quality homework time.
  2. Systematic content management: Parents should proactively participate in selecting their children’s screen content rather than passively accepting it. They can consult lists of age-appropriate, high-quality content resources recommended by professional organizations (e.g., pediatric societies, education departments). Utilize built-in or third-party content rating, filtering, and parental control features on electronic devices to restrict children’s exposure to unsuitable content (e.g., violence, pornography, excessively commercialized information). Periodically review with the child the content they have recently watched or played, discussing its value and potential impact, thereby cultivating their critical thinking and media discernment abilities.
  3. Structured behavioral contracts and positive reinforcement: For children who have attained a certain cognitive level (typically school-aged and older), collaboratively discuss and formulate family electronic product use rules, establishing a written or verbal “behavioral contract.” These rules should be specific, clear, actionable, and include a transparent system of rewards and consequences. Rewards should primarily focus on encouraging self-control, adherence to rules, and participation in a diverse range of non-screen activities, rather than simply offering more electronic product use as a reward.
  4. Promotion of positive alternative activities: Merely restricting electronic product use without providing appealing alternative activities is often ineffective. Parents and educators should actively create and offer a rich variety of non-screen activity options that align with children’s interests and developmental needs. This includes, but is not limited to: encouraging at least 60 minutes of moderate-to-vigorous physical activity daily, such as outdoor running, ball sports, or swimming, which not only benefits physical health but, as research by Caurín (31) suggests, may also help alleviate tic symptoms; cultivating at least one non-screen hobby, such as playing a musical instrument, drawing, engaging in crafts, reading, or playing board games; increasing quality parent-child interaction time, such as participating in household chores together, reading together, playing family games, or going on outdoor excursions; and creating more opportunities for face-to-face social interaction and cooperative play with peers.
  5. Dynamic monitoring and flexible adjustment: electronic product use management is an ongoing process, not a singular, fixed setup. Parents and professionals need to periodically (e.g., every few weeks or months) review and evaluate the implementation and effectiveness of the management strategy. This includes observing changes in the child’s symptoms, sleep quality, emotional state, academic performance, and their attitudes and behavioral patterns towards screen activities. Based on these assessment results, the management plan should be promptly and flexibly adjusted to accommodate the child’s growth and evolving life situations (e.g., holidays, periods of academic stress).

### 4.3 The importance of parental guidance and collaborative intervention

In managing electronic product use among children with Attention Deficit Hyperactivity Disorder (ADHD) and Tic Disorders (TDs), parents play an irreplaceable core role, while effective collaboration among family, school, and medical professionals is a key guarantee for the successful implementation of management strategies.

Enhancing parents’ media literacy and empowering their management capabilities is a primary task. Parents not only need to fully understand different types of electronic products, content, and their potential positive and negative impacts on children with ADHD/TDs, but also need to master effective communication skills, behavior shaping strategies, and boundary-setting methods. Research by Bringer (49) highlighted the internet as an important channel for parents to access ADHD-related information and peer support, but also pointed to the challenges of information overload and discerning information authenticity. Therefore, clinicians and educators have a responsibility to provide parents with authoritative and accurate sources of knowledge and guide them on how to screen and apply this information. Chen (50) developed and validated the “Parental Self-Efficacy Scale for Managing Children’s Online Risk Behaviors,” which suggests that enhancing parental self-efficacy—their confidence in successfully managing their child’s electronic product use behavior—is an important intervention target. This can be achieved through parent training workshops, individual counseling, and by providing easy-to-understand and operational guidance manuals.

Parental role modeling cannot be ignored. Children largely learn behavioral patterns through observation and imitation. If parents themselves are excessively engrossed in electronic devices or exhibit a lack of control over electronic product use in front of their children, their management demands on their children often lack persuasiveness. Research by Güzel (46) also indicated that negative media role modeling by mothers and neglect of children’s electronic product use are factors associated with problematic electronic product use in children. Therefore, parents need to reflect on and adjust their own electronic product use habits, striving to create an overall more balanced and healthy electronic product use atmosphere within the family.

Establishing a positive parent-child relationship and open communication is the foundation of effective management. Harsh prohibitions and frequent conflicts often lead to resistance from children, who may even turn to more covert electronic product use. Ideal management should be based on mutual understanding, respect, and trust. Parents should try to understand their child’s needs and motivations for using electronic products (e.g., socialization, entertainment, learning), discuss the pros and cons of electronic product use on an equal footing with the child, collaboratively establish family rules, and encourage the child to proactively seek parental help when encountering online difficulties (e.g., cyberbullying, exposure to inappropriate information). This cooperative management approach is more conducive to cultivating children’s media literacy and self-management skills.

Collaborative intervention involving family, school, and medical professionals is key to achieving long-term management success. Schools should integrate media literacy education and internet safety education into their health curriculum, guiding students to correctly understand and use digital technology, and work closely with families to jointly monitor and manage students’ electronic product use behavior. When diagnosing, assessing, and formulating treatment plans for children with ADHD/TDs, clinicians and psychotherapists should include electronic product use as a routine assessment dimension to comprehensively understand their usage patterns, duration, content preferences, and related problems (e.g., sleep disorders, emotional distress, academic impact). Based on this, they should provide families with professional, individualized screen management advice and behavioral intervention guidance. For children with severe problematic internet use or gaming disorder, referral to specialized behavioral addiction treatment facilities may be necessary. A web application proposed by Meyer (51) aimed at promoting family-school-medical collaboration, although its research subjects were more broadly children with neurodevelopmental disorders, its advocated concept of using a technological platform to promote information sharing, goal alignment, and continuous communication holds important referential value for the comprehensive management of electronic product use in children with ADHD/TDs. Only when family, school, and medical professionals reach a consensus, fulfill their respective roles, and cooperate closely can a healthy growth environment be constructed for these special children, enabling them to enjoy the benefits of the digital age while effectively mitigating its risks.

## 5 Discussion

This review, through a systematic review and analysis of existing PubMed literature, has explored the core issues surrounding electronic product use by children with Attention Deficit Hyperactivity Disorder (ADHD) and Tic Disorders (TDs). The findings reveal the complexity, multidimensionality, potential mechanisms, and variability in the extent of impact of electronic products, as well as the necessity and strategies for scientific management. It is particularly important to emphasize that the core insight from current research evidence is that the management of electronic products for children with ADHD/TDs has critically shifted from a simple “to watch or not to watch” dichotomy to a more refined and scientific approach of “how to watch well” and “how to use well.” This does not negate the potential risks of electronic product use but rather underscores the importance of maximizing benefits and minimizing harm through scientific management and guidance.

### 5.1 Clinical implications: a framework for scientific management

A primary insight from this review is that a clear hierarchical structure exists among management principles, based on the strength and consistency of the available evidence. For clinical guidance, we propose the following ranking of importance: content selection (‘what to watch’) > usage patterns (‘how to watch’) > total duration (‘how long to watch’). The consolidated evidence consistently demonstrates a significant divergence between the negative impacts of passive, high-stimulation content and the neutral or beneficial effects of interactive, structured, or therapeutic applications. This principle is uniquely capable of resolving the core paradox in the literature: why observational studies on general electronic product use show harm, while RCTs on specific digital tools reveal therapeutic potential. The finding by (6) that passive video viewing was associated with ADHD risk while interactive video was not, further solidifies the view that content is the primary determinant of a positive or negative outcome (6). Therefore, clinical guidance should pivot from a primary focus on duration limits to a more foundational emphasis on systematic content management.

(1) “What to watch”: Refined consideration of content selection is fundamental. The primary core of scientific management lies in meticulous content selection. Parents and professionals must profoundly recognize that different types of electronic content can have vastly different impacts on children with ADHD/TDs. This is far more critical than merely controlling total usage time, as appropriate content may yield benefits even within a certain duration, whereas inappropriate content can produce negative effects even with brief exposure. Recommended content types or those for use under guidance (the “positive list”) primarily include: (a) Structured cognitive training and digital therapeutic applications, such as AKL-T01 (EndeavorRx™) (27) and KAD_SCL_01 (16), which aim to improve attention and specific cognitive functions through adaptive algorithms or AI-driven stimuli. (b) Serious games and behavioral intervention tools, like “The Secret Trail of Moon (MOON)” (20) and XTics (17), used to improve cognitive abilities in children with ADHD and reduce tics through gamified ERP principles, respectively. (c) Specific types of educational and learning software, especially those that are well-designed, highly interactive, and can stimulate active thinking and problem-solving skills, such as interactive apps assisting with mathematics or reading, whose relative safety is indirectly supported by some research (6). (d) Exergaming and apps promoting physical activity, as shown in research by Benzing and Schmidt (15), which combine electronic product use with physical activity and contribute to improving executive functions (EFs) and motor skills. (e) Creative and expressive applications, such as digital painting, music creation, and introductory programming, which provide platforms for children to express themselves and create, fostering logical thinking. (f) Guided social interaction and support platforms (mainly for adolescents), such as participating in disorder-related support communities (49), using assistive applications like Kiddo (52), and internet-based Cognitive Behavioral Therapy (iCBT) like BIP TIC or iHRT (32, 33). The prerequisite for all these beneficial or neutral applications is their use under professional assessment and guidance, or effective family management. Content types to be strictly limited or avoided (the “negative list”) include: (a) High-intensity stimulation, fast-paced, violent, or terrifying content, which is directly related to symptom exacerbation (13). (b) Passive viewing, low-interactivity, low-nutritional-value entertainment content, which not only encroaches on beneficial activity time but may also solidify poor usage habits (6). (c) Games with addictive designs or gambling mechanisms, to which children with ADHD/TDs are more susceptible to dependence (25, 26). (d) Social media abuse that easily triggers social anxiety, comparison, or cyberbullying, potentially aggravating emotional problems (8, 42). (e) Content purely for commercial marketing purposes or filled with numerous advertisements. When performing such content screening, parents and professionals need to recognize that even content from the “positive list” is not a case of “the more, the better”; it still requires comprehensive management combined with the principles of “how long to watch” and “how to watch.”

(2) “How long to watch”: Scientific principles and strategies for duration control. After establishing the importance of content selection, the scientific management of electronic product use duration is equally indispensable. The foremost principle is the prioritization of core developmental activities; that is, the use of electronic products must not encroach upon a child’s essential daily sleep, sufficient physical activity, quality academic time, and face-to-face social interactions with family and peers. With this prerequisite, defining a “moderate” range is a dynamic and multidimensional issue. A pragmatic starting point is to reference general recommendations for recreational electronic product use in children and adolescents (e.g., <2 hours/day for school-aged children) as a baseline (44, 48), although this should not be viewed as a rigid rule. For children with ADHD and Tic Disorders, this threshold requires highly individualized adjustment based on specific contexts. For instance, age is a critical contextual factor, and restrictions should be far stricter for younger children (e.g., 1–3 years old), for whom research has associated even 1–60 minutes of passive viewing with an increased risk of preschool ADHD (6). Likewise, based on the child’s core symptoms, evidence suggesting that electronic product use exceeding 2 hours per day in children with Tourette Syndrome is significantly associated with sleep problems indicates that more stringent limits may be necessary for those with sleep disturbances or TDs (11).

However, the key to elevating duration management to a scientific standard lies in moving beyond fixed numbers to establish a flexible adjustment mechanism based on content quality and individual symptom feedback. Quantitative thresholds are inherently context-dependent: the neurocognitive impact of a one-hour session with a creative, interactive application is not equivalent to that of one hour of a high-stimulation, fast-paced video game. Therefore, the “time budget” can be more elastic for higher-quality content. Ultimately, and most importantly, the golden standard for “moderation” must be the individual child’s clinical presentation. If a child’s tics, inattention, or sleep patterns worsen, their current level of use—regardless of how low it may seem—is, by definition, no longer “moderate” for them. This necessitates that clinicians guide parents in continuous monitoring and be prepared to adjust limits based on these observations. This principle aligns with the evidence that “problematic use” is a better predictor of ADHD symptoms than mere total duration (7), emphasizing that the core of management should shift from “counting minutes” to “observing and responding to the child’s state” to find a truly scientific and appropriate boundary for each individual child.

(3) “How to watch”: Refined management of usage patterns. Merely controlling duration and selecting content is insufficient; how electronic products are used—that is, the refined management of usage patterns—also profoundly affects the final outcome. This primarily includes: (a) Emphasizing active participation and interactivity. Encourage children to engage in screen activities that require active thinking, creation, and problem-solving, rather than prolonged passive viewing (6). (b) Optimizing the usage context, i.e., “screen zone” and “electronic product use slot” management. Promote “screen-free bedrooms” (9, 10), “use in common areas,” “screen-free dining tables,” and “screen-free family time,” and strictly enforce “no screens before bed” (8). (c) Enhancing the quality of parental mediation, including active mediation/co-use, restrictive mediation (46, 50), and technical mediation. (d) Cultivating media literacy. Parents should first improve their own literacy and then guide children to develop digital information discernment skills and online risk identification abilities. (e) Encouraging a balanced “digital nutrition menu,” avoiding prolonged singular immersion in one specific type of screen content. (f) Focusing on the transfer and application from screen to reality. Encourage children to apply the knowledge and skills learned on screen to real life. Through such refined content selection, duration control, and usage pattern management, combined with individualized assessment and collaborative support from family, school, and medical professionals, it is possible to more scientifically guide children with ADHD/TDs towards healthy development in the digital age.

(4) Beneficial applications of electronic products under specific conditions: Evidence-based outlook and examples. The following table (**Table 3**) summarizes some digital tools or methods that have shown potential for children with ADHD/TDs in research, aiming to provide a reference for clinical practice and families. It needs to be emphasized that the application of these tools is mostly recommended under professional guidance and as part of a comprehensive intervention plan, rather than as a standalone solution.

**Table 3:** Examples of digital tools/methods with potential benefits for children with ADHD/TDs, their evidentiary basis, and key management points.

Digital tool/method category	Specific name/example (Literature source)	Target disorder	Main intervention goal/mechanism	Key research findings/evidence	Core management & usage recommendations
Digital Therapeutics (Gamified Cognitive Training)	AKL-T01 (EndeavorRx)	ADHD	Improve objective attention, reduce functional impairment via adaptive multitasking game	Improved TOVA Attention Comparison Score (ACS); in-game cognitive metrics correlated with clinical improvement (27)	Prescription use; follow medical advice (e.g., ~25 min/day, 5 days/week, typically 4–8 weeks); monitor adherence and potential minor adverse reactions (e.g., headache, frustration).
Serious Games (Cognitive/Emotional Regulation)	The Secret Trail of Moon (MOON)	ADHD	Improve emotion regulation, working memory, inhibitory control, material organization via cognitive training game	Observed improvements in working memory, inhibitory control, and material organization in highly engaged patients (20)	Recommended as a supplement to multimodal therapy; focus on individual engagement and interest; parental assistance may be needed to ensure completion of training as scheduled.
Gamified Behavioral Intervention	XTics	TDs	Reinforce tic suppression behavior via instant positive feedback (based on ERP principles)	Significantly prolonged inter-tic intervals, reduced YGTSS-TTS and Rush scores; parent-reported effects sustained for 3 months (17)	Daily play; initial professional guidance for setup may be needed; high adherence is key; effective supplement or alternative to traditional behavioral therapy, especially for those unwilling/unable to access traditional face-to-face therapy.
Exergaming	(General term, e.g., specific cognitive engagement-type exercise games on platforms like Kinect, Wii Fit)	ADHD	Improve executive functions (inhibition, switching), motor skills; combines physical activity with cognitive engagement	8-week intervention (3 times/week, 30 min/session) significantly improved inhibitory control, task-switching reaction time, motor skills, and overall psychopathology in children with ADHD (15)	Select games appropriate for age, interest, and ability; ensure safe activity space to prevent injuries; regular participation; still need to control total electronic product use time, avoid replacing real outdoor activities and social interaction.
Digital Cognitive Stimulation Program	KAD_SCL_01	ADHD	AI-driven personalized cognitive stimulation to improve inhibitory control, visuospatial working memory	After 12 weeks of training, KAD_SCL_01 group performed significantly better on inhibitory control and visuospatial working memory tasks than control (commercial video game); associated with changes in posterior brain alpha wave activity (16)	Professional assessment may be needed to determine suitability; train according to recommended protocol (e.g., 3 days/week, 15 min/day, for 12 weeks); monitor for potential overstimulation or frustration with behavioral observation and feedback.
Internet-based Cognitive Behavioral Therapy (iCBT)	BIP TIC (Internet-based Exposure and Response Prevention); iHRT (Internet-based Habit Reversal Training)	TDs/TS	Provide standardized ERP or HRT, increasing treatment accessibility	iCBT is comparable or non-inferior to face-to-face therapy in reducing tic severity; remote therapist support can improve adherence and outcomes (32, 33)	Typically requires therapist (remote) support and guidance to ensure treatment fidelity; family cooperation needed for offline practice and behavioral records; address internet accessibility and technical operation issues; focus on child and family motivation and sustained engagement.
Adjunctive Educational & Life Skills App	(GaKiddo mified adjunctive therapy web application)	ADHD	Gamified approach to improve quality of life, provide ADHD-related knowledge and management tools for children with ADHD	After 2 months of use, Conners’ Parent Rating Scale scores significantly decreased, indicating improved quality of life (52)	As an adjunctive support tool, cannot replace core medication or behavioral therapy; parents need to participate in selection and supervision, ensure content is scientific, age-appropriate, and coordinated with the overall treatment plan.
Virtual Reality (VR) Attention Training	(Specific VR attention training games/programs, e.g., VR systems for ADHD mentioned in literature)	ADHD	Provide immersive attention tasks in simulated environments to train sustained and selective attention	Some studies show positive effects of VR cognitive training on attention in children with ADHD, but evidence quality and long-term effects need more high-quality research validation (18)(mentioned in review)]	Higher technical equipment requirements; monitor potential side effects (e.g., motion sickness, visual fatigue); ensure scientific and reasonable content design, avoid overstimulation; typically used short-term under professional guidance.

### 5.2 Prudent interpretation of evidence and real-world considerations

When applying these strategies, a scientifically prudent approach is essential. First, the finding that a large number of commercial digital tools lack evidence-based support is not an isolated phenomenon. This disconnect between the commercial market and scientific evidence is a recurring and critical issue in the field that requires urgent attention. For example, a systematic review by Păsărelu et al. found that among 109 ADHD-related apps in application stores, almost none provided evidence of their efficacy (22). Similarly, in a scoping review of ADHD applications over the past decade, Hernandez-Capistran found that only two of 35 commercial apps had received endorsement from a medical institution (53). Second, the potential benefits of digital tools should not be overgeneralized. As summarized in **Table 2**, much of the causal evidence for the benefits of digital therapeutics comes from small-scale randomized controlled trials, which are often industry-funded or conducted within narrow age ranges (15, 17). When interpreting these results, it is imperative to consider potential conflicts of interest and to recognize that the long-term effectiveness of these tools and their generalizability to more diverse clinical populations still require validation through further independent research. Clinicians and families should be guided to view these applications as components within a comprehensive treatment plan, rather than as standalone solutions that can replace core therapies.

Furthermore, it is crucial to acknowledge that the feasibility of the management recommendations proposed in this review is not uniform across all families. Households with limited socioeconomic resources may face significant obstacles, such as insufficient parental supervision due to demanding work schedules, a lack of financial capacity to purchase high-quality paid applications or stable internet services, and limitations in parental digital literacy. Therefore, clinical guidance and public health strategies must be sensitive to real-world contexts, prioritizing low-cost, high-impact strategies. This could include emphasizing the establishment of firm “screen-free” household rules (e.g., during mealtimes and in bedrooms), promoting free, high-quality educational resources provided by public institutions (such as libraries and public television), and fostering community-based support networks for parents to share effective, low-cost management experiences.

### 5.3 Limitations of the review

Although this review aimed to be comprehensive and systematic, several limitations should be acknowledged. First, due to the significant heterogeneity in the design (predominantly observational) and methodology of the included studies, this review employed a narrative synthesis rather than a quantitative meta-analysis. While this approach allowed for the integration of more diverse evidence, it also limits our ability to draw definitive conclusions about the magnitude of specific effects. Moreover, the vast majority of included studies were of cross-sectional or observational design; while capable of revealing important associations between variables, they cannot establish clear causal relationships. Second, as with all literature reviews, the results may be subject to potential publication and language selection biases. Finally, we did not systematically extract or structurally compare data across different subgroups (e.g., different age groups, comorbid conditions, medication status). This was primarily due to the significant heterogeneity in reporting methods, variable definitions, and subgroup analyses within the primary studies themselves. Consequently, much of the key subgroup information was either missing or not directly comparable, which limited the ability of this review to draw stable, generalizable conclusions about differential effects in specific subgroups. Collectively, these limitations serve as a reminder that conclusions must be applied with careful individual consideration in clinical practice and highlight the necessity for more rigorous longitudinal and experimental research in the future.

## 6 Conclusion and future directions

### 6.1 Conclusion: translating scientific management into a clinical pathway

For children with Attention Deficit Hyperactivity Disorder (ADHD) and Tic Disorders (TDs), the management paradigm for electronic product use should transition from simplistic “restriction” or “prohibition” to scientific “guidance and management.” The core conclusion of this review is that the crux of management has shifted from a simple “to watch or not to watch” dichotomy to the implementation of a refined, scientific management strategy centered on the hierarchy of: content selection > usage patterns > total duration.

To translate “scientific management” from an abstract concept into concrete practice for clinicians and families, a clinical pathway composed of four stages can be followed: “Assess-Plan-Implement-Monitor.” The pathway begins with comprehensive Assessment and Documentation: clinicians can guide parents to use a “screen-time diary” or built-in device functions to record baseline usage, thereby analyzing and identifying high-risk content and patterns, and to communicate with the child to assess their motivations for use. Based on this, the second step is Collaborative Planning, which involves setting core goals with the family, such as “safeguarding core developmental needs,” creating a “Family Media Use Plan” using tools from organizations like the American Academy of Pediatrics (AAP), and formulating specific content substitution strategies to gradually replace high-risk activities with “green-light content.” The third step is Stepwise Implementation, emphasizing gradual change, starting with the most achievable goals, and proactively providing appealing non-screen alternatives, while parents lead by example. Finally, the fourth step is Continuous Monitoring and Adjustment, where the management plan is flexibly adjusted by periodically reviewing progress and observing changes in symptoms and behavior to adapt to the child’s growth and needs at different times. For a set of practical tools and templates to aid in this process, please refer to Appendix 1 (see **Supplementary Materials**).

### 6.2 Future directions

While systematically reviewing existing evidence, this review also reveals several critical gaps in the current research landscape. To further advance the scientific understanding and effective management of electronic product use in children with ADHD/TDs, future research should be deepened and expanded in the following areas:

Deepen Longitudinal and Multimodal Research on Impact Mechanisms:
  Longitudinal Neuroimaging Studies: Current research is predominantly cross-sectional, which precludes causal inference. There is an urgent need for long-term longitudinal cohort studies that combine techniques such as functional magnetic resonance imaging (fMRI) and electroencephalography (EEG). Such studies should track the screen exposure trajectories of children with ADHD/TDs from an early age to dynamically observe corresponding changes in their brain structure, functional connectivity (especially fronto-striatal circuits), and neurotransmitter systems (such as the dopaminergic pathways), thereby elucidating the long-term causal effects of different electronic product use patterns (content, duration, interactivity) on neurodevelopment.
  Multimodal Data Integration: Future research should integrate neuroimaging, genetic data, behavioral assessments, physiological indicators (e.g., heart rate variability, cortisol levels), and Ecological Momentary Assessment (EMA) data. This would allow for the construction of multilevel analytical models to more comprehensively reveal how individual susceptibility (genetics) and environmental exposure (electronic product use) interact to influence symptoms and cognitive functions in ADHD/TDs.
Explore Refined “Dose-Response” Relationships and Differential Impacts:
  Parameterized Studies of Content and Patterns: Moving beyond broad categories like “games” or “videos,” future experimental studies should randomly assign subjects to groups exposed to screen content with varying parameterized characteristics (e.g., fast *vs*. slow pace, high *vs*. low interactivity, reward frequency, presence of violence/suspense). This would allow for the precise quantification of the immediate and short-term impacts of different content elements on symptoms, cognition, and emotion.
  Research on Differential Effects in Subgroups: Studies should be specifically designed to compare the effects of electronic products on different subgroups (e.g., different ADHD presentations, presence of comorbid OCD/anxiety, different age groups, medication status). For instance, a study could specifically compare the physiological and behavioral responses of ADHD children with comorbid sensory processing disorders when exposed to high- versus low-stimulation screen content.
Optimize and Validate Digital Interventions:
  High-Quality Randomized Controlled Trials (RCTs): For promising digital therapeutics (e.g., AKL-T01, XTics) and serious games, more large-scale, multi-center RCTs with active control groups (e.g., standard behavioral therapy, other types of games) and long-term follow-up are needed. These trials are essential to definitively establish their efficacy, mechanisms of action, optimal “dosage” (frequency and duration of use), and the durability of long-term effects.
  Development of Personalized and Adaptive Interventions: Leverage machine learning and artificial intelligence to develop personalized digital intervention tools that can dynamically adjust difficulty and strategies based on the user’s real-time performance (e.g., in-game data, physiological feedback), thereby maximizing training effects and user engagement.
  Combination Intervention Research: Explore the synergistic effects of combining digital interventions with traditional treatment methods (e.g., medication, behavioral therapy). For example, research could investigate whether digital cognitive training can enhance the effects of medication on executive function improvement.
(4) Evidence-Based Construction of Collaborative Management Models:
  Development and Validation of Family Intervention Programs: Develop and evaluate structured media literacy education and screen management training programs for families with ADHD/TDs (e.g., Parental Media-related-skill Training, PMT). RCTs should be used to validate the effectiveness of these programs in improving children’s electronic product use behaviors, parent-child relationships, and parental self-efficacy in management.
  Construction and Evaluation of Collaborative Platforms: Design and evaluate multi-party collaborative digital platforms, as advocated by Meyer (51), to test their practical utility in promoting information sharing, goal alignment, and intervention consistency among families, schools, and healthcare providers.
  Development of Evidence-Based Clinical Guidelines: After sufficient high-quality research evidence has been accumulated, academic societies should undertake the development of evidence-based clinical practice guidelines specifically for children with ADHD/TDs. These guidelines should cover content recommendations, duration advice, risk warnings, and management strategies to provide authoritative, actionable guidance for frontline clinicians and families.

Finally, researchers and policymakers must confront and address implementation barriers. Even with perfect guidelines, real-world obstacles such as cost, accessibility, and the demands on parental time and training may hinder their adoption in families with limited socioeconomic resources. Future research should focus more on implementation science to develop and validate low-cost, easily disseminable, and culturally sensitive management strategies, ensuring that all children with special needs can benefit from healthy development in the digital age.
