# Supplementary material for: The impact of electronic product use on children with tic disorders and ADHD, and management strategies: a review
Source: Front Psychiatry. 2025 Oct 21;16:1665047. doi: 10.3389/fpsyt.2025.1665047 (PMC12583088; doi:10.3389/fpsyt.2025.1665047)
Supplement: Supplementary file 1 [file DataSheet1.pdf]

Appendix 1: Practical Management Toolkit

Category	Specific Tools/Resource Examples	Purpose and Use
Assessment & Recording Tools	Screen-Time Diary	A templated form to help parents systematically record a child's weekly screen use details (time, content, context) for baseline assessment and problem identification.
	Built-in Device Functions (e.g., Apple Screen Time, Google Family Link)	Automatically tracks device usage time and app usage, providing objective data support.
Planning Tools	American Academy of Pediatrics (AAP) Family Media Plan Online Tool (healthychildren.org)	An interactive online tool that guides family members to discuss and create a personalized, printable family media use agreement.
	Parent-Child Behavior Contract Template	A printable contract template that clearly defines screen use rules, responsibilities, and consequences for breaking rules, adding seriousness to implementation.
Content Screening & Guides	Common Sense Media (commonsensemedia.org)	Provides age-appropriateness ratings, content analysis (violence, language, etc.), and educational value assessments for movies, games, apps, and books to help parents make informed choices.
	Children's Technology Review	Provides independent, professional reviews of digital products for children.
Technical Management Tools	Parental Control Software (e.g., Qustodio, Net Nanny)	Allows parents to set time limits, filter inappropriate content, block specific applications, and track location on their child's devices.
Therapeutic Digital Tools	EndeavorRx (by Akili Interactive)	An FDA-approved, video game-based prescription digital therapeutic designed to improve attention function in children with ADHD.

Category	Specific Tools/Resource Examples	Purpose and Use
	XTics	A gamified behavioral intervention tool, based on research, used to manage tic symptoms.
